# Lost in Transition: Delayed Diagnosis of Autism Spectrum Disorder Following Early Migration

**DOI:** 10.7759/cureus.82883

**Published:** 2025-04-24

**Authors:** Tiago Aguiar Soares, Mariana Neves, Rita Penha, Daniela Couto

**Affiliations:** 1 Department of Child and Adolescent Psychiatry, Local Health Unit of Western Lisbon, Lisbon, PRT

**Keywords:** autism spectrum disorder, cultural barriers, delayed diagnosis, developmental delay, high-functioning autism, school refusal, sensory processing difficulties

## Abstract

We present the case of a 16-year-old male whose diagnosis of autism spectrum disorder (ASD) was significantly delayed due to the masking effect of early migration. At the age of five, during a critical window for neurodevelopmental identification, he migrated from Portugal to the United Kingdom following an eight-month separation from his primary caregiver. In the years that followed, early autistic features such as language regression, sensory sensitivities, and social withdrawal were attributed to cultural adjustment and second-language acquisition.

A comprehensive retrospective developmental assessment in adolescence ultimately revealed that these behaviours were not solely adaptive responses but reflected longstanding features of ASD. This case underscores how environmental transitions during key developmental periods may hinder early identification of neurodevelopmental conditions. It highlights the need for meticulous developmental history-taking and culturally sensitive assessment, particularly when evaluating migrant children whose presentation may be shaped by both intrinsic vulnerabilities and external contextual factors.

## Introduction

Autism spectrum disorder (ASD) is a complex neurodevelopmental condition characterized by persistent deficits in social communication and interaction, alongside restricted, repetitive patterns of behavior and interests [1]. Although the clinical features of ASD are often recognisable from early childhood, the subtler manifestations in individuals without intellectual disability and with intact or mildly impaired functional language frequently go unnoticed, particularly in complex psychosocial contexts [2]. They often struggle with pragmatic communication, emotional reciprocity, sensory integration, and behavioural flexibility. These features, while clinically significant, may be misinterpreted as personality traits, anxiety, or social inhibition, especially in children who otherwise show good verbal fluency and academic potential [1,2].

The diagnostic process for ASD is particularly complex in children from migrant backgrounds, where cultural and linguistic factors intersect with developmental trajectories [3-5]. Migration during early childhood introduces significant environmental changes, including language acquisition, adaptation to new educational systems, and cultural differences in behavioral expectations. These factors can obscure early signs of ASD, such as delays in social communication or sensory hypersensitivities, which may be misinterpreted as consequences of environmental stress rather than neurodevelopmental issues [3,4].

Cultural norms and parental expectations also play a significant role in the recognition of ASD. In some cultures, behaviors such as avoiding eye contact or exhibiting intense focus on specific activities may be seen as shyness or diligence rather than developmental red flags [5]. Additionally, healthcare systems in host countries may lack the cultural competence needed to evaluate diverse populations effectively. Studies show that families from immigrant backgrounds often experience delays in accessing diagnostic services due to language barriers, cultural misunderstandings, and systemic inequities [4,5].

This case report presents a 16-year-old male whose diagnosis of ASD was significantly delayed following early migration from Portugal to the United Kingdom. His initial presentation - marked by sensory sensitivities, restricted interests, and social withdrawal - was interpreted as a transient adjustment reaction. It was only through a comprehensive developmental re-evaluation in adolescence that a clear diagnostic picture emerged. This report illustrates how these diagnostic complexities can unfold in a real-world context, highlighting the importance of culturally informed developmental assessments, especially when ASD presents with subtle features and occurs in the context of environmental disruption.

## Case presentation

A 16-year-old male was referred to our outpatient service due to persistent school refusal, severe anxiety, and social withdrawal, which had escalated over the previous three years. These symptoms significantly impacted his academic engagement and family life. The referral prompted a comprehensive neurodevelopmental evaluation.

The patient lived with his mother and six-year-old brother. His parents separated when he was two years old, after a conflict-ridden relationship. He was primarily raised by his mother and maternal grandmother. He had minimal contact with his biological father, which he described as a source of sadness, recognising no effort was made by either to maintain the relationship. The mother had two significant life partners afterwards, with whom the patient was very close. However, he abandoned the family, which hurt him significantly. The second partner was his brother’s father, whom he rejected, contributing to their early separation.

His relationship with his mother was emotionally intense and ambivalent, marked by dependency and expressions of anger towards her. The mother describes herself as overprotective, particularly after experiencing anxiety and intrusive thoughts during the postpartum period. She recognises that her parenting may have restricted her son's autonomy (e.g., refusing to let him ride a bicycle due to intrusive fears that he may break his skull). Besides this, the family history includes maternal attention deficit and hyperactivity disorder (ADHD) but no other confirmed mental health issues.

He had a previous follow-up by a Child and Adolescent Psychiatry Consultant in the UK and was treated with sertraline, titrated to 150 mg for six months, with mild improvement. A social prescriber also supported him, and psychotherapy was proposed but not initiated, as he would not engage with most professionals.

His early developmental milestones, such as gross motor skills, were achieved within typical timeframes. However, subtle signs of neurodevelopmental divergence were evident from early childhood. These included sensory hypersensitivities, restricted interests, and atypical social interactions. He displayed rigid preferences for certain toy details and activities, significant concern about cleanliness, and limited adaptability in play with peers. He showed a strong attachment to certain toys and was notably insistent on keeping them perfectly clean. He also needed to have his hands always clean and insisted every night that his grandmother wash the bathtub with detergent before his bath.

He also exhibited a strong preference for repetitive media exposure, rewatching *Madagascar* repeatedly, and demonstrated precocious interests in flags, chess, fossils, and animals - memorising detailed information beyond developmental expectations (e.g., by age four, he recognised most of the world’s flags). His language development was delayed, with his first words around 24 months. Although early vocalisations and a social smile were present, his non-verbal communication remained atypical: he did not point to indicate, never nodded or waved, and relied exclusively on verbal responses to communicate affirmations. He consistently used multiple idiosyncratic neologisms (more than five), one of which persisted for several years and showed deferred echolalia. Few stereotyped behaviours were observed (e.g., celebrating with hands near the face, tiptoeing around age 10 for a few months), with no consistent mannerisms.

His temperament was described as easygoing, calm, and sociable. He did not have tantrums and rarely cried before age two, except when ill. He appeared tolerant of frustration until around age five, when defiance began to emerge. This tendency escalated after migration: by age seven, he began to show aggression when frustrated, directed exclusively towards family members (e.g., in response to being asked to eat with the family, complete homework, or attend school). His mother reported giving up on verbal confrontations after this point. He felt the need to co-sleep for a significant part of his childhood and began experiencing initial insomnia from age five, which has persisted.

He began attending kindergarten at 18 months and exhibited no significant separation anxiety. He had two close friends, enjoyed playing with toy cars and tricycles, and engaged in pretend play (e.g., acting as a doctor or a monster), though he had poor adaptability when peer interests diverged. In a small, affectionate preschool setting, he preferred younger children and maintained a limited social circle (one to two peers). In public spaces, he avoided unfamiliar children and required adult mediation to initiate play.

At age five, the patient migrated from Portugal to the United Kingdom, following an eight-month separation from his mother, who migrated ahead. The relocation brought significant environmental, linguistic, and cultural shifts. He experienced language regression during the first three months, avoiding verbal communication outside the home while acquiring English. Upon school entry in the UK, his affective and social behaviours previously accepted (e.g., hugging other children and the teachers) were labelled inappropriate by educators, initiating a pattern of misunderstanding and social exclusion. He reported feeling different from his peers. He described boys as “savages” and consistently preferred the calmer, more predictable company of girls or younger peers.

Educators and caregivers attributed his behaviours to cultural and linguistic adjustment, delaying neurodevelopment assessment. Sensory aversions and rigid routines also intensified. He began refusing to eat with others, reporting sensory overload when watching or hearing others eat. He only accepted “safe foods” (e.g., plain rice/pasta, grilled meat without sauces or extras) and started eating alone in his room. These aversions contributed to school refusal, particularly during lunch hours.

By adolescence, the patient presented with severe social anxiety, persistent sensory hypersensitivities, and school refusal. These difficulties became prominent in seventh grade and persisted, causing significant social and academic impairment. He reported rare but intense panic attacks triggered by the anticipation of social interaction or overstimulating environments.

Sensory sensitivities remained a dominant feature, with pronounced aversion to textures (e.g., sauces), sounds (e.g., chewing, barking), and visual stimuli (e.g., cutting tomatoes). He avoided toothbrushing for years and demonstrated tactile-seeking behaviours such as licking metal and touching rough surfaces or animal fur.

Social interactions were severely restricted. He avoided peers, particularly adolescents, describing them as “horrible.” He preferred online friendships and felt more comfortable interacting with younger children, noting they were easier to understand. Although he expressed a desire for connection, he lacked social reciprocity and conversational flexibility. His speech was dominated by restricted interests, mainly *Pokemon®* and manga, and he questioned the utility of small talk. Greeting behaviours (verbal and non-verbal) were minimal or absent.

His restricted interests were pervasive and longstanding. His bedroom was described as a “museum” of *LEGO®* and *Pokemon®* collectables. He has been consistently focused on *Pokemon®* since age four and maintains his *LEGO® *constructions in a specific order, becoming distressed if disturbed. His drawing always starts with a detail, and the figure must face right. He also played video games on multiple devices for over five hours daily, often late into the night, contributing to poor sleep hygiene and screen dependency. These behaviours, while sometimes comforting, further limited his flexibility and social engagement.

A formal diagnostic process was initiated, incorporating a comprehensive developmental history, direct clinical observations, and standardised screening tools, including the Social Responsiveness Scale - Second Edition (SRS-2), Autism Spectrum Screening Questionnaire (ASSQ), Social Communication Questionnaire (SCQ), and Youth Self-Report (YSR). Additionally, the Autism Diagnostic Interview-Revised (ADI-R) was administered.

Based on the integration of these data, the clinical formulation supported the International Classification of Diseases, 11th Revision (ICD-11) diagnoses of ASD without disorder of intellectual development and with mild or no impairment of functional language (6A02.0); social anxiety disorder (6B03), with panic attacks as a prominent associated feature, and gaming disorder, predominantly online (6C51.0) [6].

Management and treatment

A comprehensive multidisciplinary plan was implemented to address his complex clinical needs, including pharmacological, behavioural, educational, and familial interventions.

In December 2023, he was initiated on fluoxetine 10 mg/day, titrated by 10 mg every two weeks up to 50 mg/day, using the oral solution formulation due to swallowing aversion. This treatment led to improvements in mood stability and reduction of social anxiety symptoms. In parallel, melatonin 2 mg at night was prescribed to manage initial and intermittent insomnia.

Given the persistence of anxiety symptoms, gabapentin was later introduced and titrated to 300 mg/day, resulting in modest improvement. However, it was subsequently discontinued due to limited overall benefit.

Behavioural therapy focused on gradual exposure and anxiety management. He engaged in 20 cognitive behavioural therapy (CBT) sessions over six months with a licensed psychologist. The program targeted cognitive distortions and included exposure to anxiety-inducing settings (e.g., school). A structured reintegration plan addressed school refusal. It began with visits during off-hours and then gradually progressed to classroom attendance. The patient’s educational needs were addressed through collaboration with his school to create an individualised education plan (IEP). Adjustments included access to a quiet zone, being able to wear noise-cancelling headphones, sensory tools and fidgets, flexibility in curriculum delivery, and regular home-school communication.

Efforts were made to broaden the patient’s social world through structured activities aligned with his interests, such as hobby-based clubs, to provide a low-pressure setting for interacting with like-minded peers. Virtual social skills training, leveraging his comfort with online communication and participation in small-group programs like the Program for the Education and Enrichment of Relational Skills (PEERS) to practice conversational skills, turn-taking, and non-verbal communication in a controlled setting.

The patient’s short-term goals included restoring school attendance and developing coping mechanisms for sensory overload. Long-term goals included improving social communication and peer relationships, and preparing for post-secondary education in game design, his area of interest.

Progress was monitored regularly by a multidisciplinary team. He resumed partial school attendance, gained better control over anxiety, and began engaging socially in low-pressure environments. His routines became more flexible, oral hygiene improved, and dietary rigidity reduced. At the time of reporting, he remained on fluoxetine (50 mg/day) and melatonin (2 mg/day).

## Discussion

Migration during early childhood significantly shaped this patient’s developmental and diagnostic trajectory. The transition from Portugal to the UK at the age of five introduced abrupt linguistic and cultural shifts as well as a temporary separation from his primary attachment figure. These changes contributed to the misinterpretation of early symptoms, obscuring the recognition of underlying neurodevelopmental differences. His language regression and social withdrawal, which emerged during second-language acquisition, were initially seen as part of a normal adaptation process. This interpretation delayed the diagnosis of ASD for over a decade.

This case illustrates a well-documented diagnostic challenge: systemic and cultural factors often contribute to delayed ASD recognition in migrant populations [3,4]. Educators and clinicians unfamiliar with cultural nuances may overlook or misattribute ASD-related behaviours [5]. In this case, features such as rigid routines, restricted interests, limited social reciprocity, and pronounced sensory sensitivities were viewed through the lens of cultural adjustment rather than as core symptoms of a neurodevelopmental condition. This aligns with literature showing that children from minority or migrant backgrounds frequently face delays in accessing diagnosis due to limited cultural competence within healthcare and education systems [3-5].

Sensory hypersensitivities were among the most functionally impairing features in this patient. His intense aversions to food textures, chewing sounds, and tactile sensations led to rigid eating habits, oral hygiene avoidance, and distress in social settings such as classrooms. These sensitivities contributed directly to school refusal and social withdrawal. Evidence supports the role of desensitisation techniques, environmental modifications, and individualised coping strategies in improving daily functioning in adolescents with ASD, highlighting the importance of targeted sensory support [7].

Restricted interests, particularly his long-standing preoccupation with *Pokemon®*, served a dual function: they provided emotional stability and predictability in an overstimulating world but also reinforced rigidity and limited social engagement [8]. Structured interventions can channel such interests into therapeutic opportunities, such as entry points in social skills training. But without appropriate guidance, they may perpetuate social isolation [8].

Additionally, the patient’s engagement in online gaming, which met criteria for gaming disorder, further contributed to social withdrawal and disrupted routines. While gaming offered a predictable and immersive environment, it also reinforced avoidance behaviours, reduced real-life social interaction, and interfered with healthy sleep patterns [8]. Interventions addressing media balance and promoting alternative sources of gratification were essential components of care.

Social withdrawal in this case was multifactorial: rooted in core ASD-related social communication difficulties, compounded by secondary social anxiety and a history of peer rejection. Over time, these experiences appeared to shape a detached relational style marked by emotional distance and interpersonal rigidity. This style likely emerged as a defensive adaptation to repeated overstimulation, social failure, and perceived rejection. While initially protective, such coping mechanisms may become reinforced over time through persistent avoidance, limited relational modelling, and immersion in solitary activities. In adolescents with ASD and intact verbal abilities but poor intuitive social understanding, these patterns may evolve into traits resembling avoidant or schizoid functioning if unaddressed [2].

Recognising and intervening in these emerging dynamics is essential not only to reduce symptom burden but also to support the development of a positive social identity, healthy self-concept, and sustainable relational strategies. This is particularly important given the known vulnerability of adolescents with ASD to bullying and social exclusion, which further fuels mistrust, emotional dysregulation, and retreat into isolative patterns [9,10].

The patient’s preference for interacting with younger children can also be understood in this context as a strategy to lower social demands and reduce anxiety. However, this adaptation may interfere with the development of age-appropriate relational skills. Structured social skills training, such as the PEERS programme, offered a safe and effective avenue for practising conversational reciprocity, turn-taking, and non-verbal communication in a controlled environment [11].

Family dynamics significantly influenced the patient’s developmental course. His close, emotionally intense relationship with his mother offered support but also contributed to dependence and avoidance of external demands. While this attachment may have buffered some early distress, it also reinforced withdrawal behaviours and limited opportunities to build autonomy. Parent-mediated interventions aimed at promoting gradual autonomy were an essential component of his management, echoing evidence that such approaches improve adaptive functioning in adolescents with ASD [12]. The absence of a consistent paternal figure, combined with early familial stress during critical developmental windows, likely added to the patient’s emotional vulnerabilities.

Educational challenges, particularly persistent school refusal and difficulty coping with standard classroom environments, required a highly individualised educational response. Accommodations such as sensory-friendly environments, flexibility in curriculum delivery, and gradual reintegration strategies were key to reducing school-related anxiety and enabling academic participation. Research supports these measures as effective in enhancing school engagement and reducing distress in students with ASD [13,14].

Close collaboration between the family and school was critical to sustaining these accommodations. Regular home-school communication allowed for the timely identification of difficulties, adjustments to support strategies, and reinforcement of therapeutic goals. Interventions targeting both academic and social domains, such as exposure-based therapy for school attendance and group-based social training, were mutually reinforcing and contributed to the patient’s global improvement.

Ultimately, this case reinforces the importance of a flexible, individualised, and multidisciplinary approach to ASD management, particularly in adolescents with subtle presentations complicated by environmental and cultural factors. Accurate diagnosis enabled the implementation of tailored interventions, which, despite the delay, resulted in meaningful functional improvement. It reinforces the need to maintain diagnostic curiosity when facing complex behaviours shaped by both neurodevelopmental vulnerability and environmental adversity. Even when identification comes late, responsive, individualised care can foster growth, autonomy, and a more hopeful developmental trajectory.

## Conclusions

This case highlights the complexities of diagnosing and managing ASD without intellectual or significant language impairment in a culturally and linguistically diverse individual. Migration and its associated environmental and cultural changes delayed the recognition of developmental red flags, complicating the diagnostic process. Sensory hypersensitivities, restricted interests, and social withdrawal were exacerbated by contextual stressors and family dynamics, necessitating a comprehensive, multidisciplinary approach. Tailored interventions addressing sensory, social, and educational challenges are essential for fostering adaptive functioning, independence, and long-term well-being.

Moreover, this case underscores the importance of cultural sensitivity and flexibility in clinical evaluations, particularly when working with individuals in vulnerable contexts such as immigrants. Adjusting assessment tools and maintaining diagnostic curiosity when behaviours seem context-driven are essential to avoid misinterpretation or delayed recognition. Even when diagnosis is late, timely and individualised intervention can improve functioning and overall quality of life.
